# Identification of Conserved and Potentially Regulatory Small RNAs in Heterocystous Cyanobacteria

**DOI:** 10.3389/fmicb.2016.00048

**Published:** 2016-02-01

**Authors:** Manuel Brenes-Álvarez, Elvira Olmedo-Verd, Agustín Vioque, Alicia M. Muro-Pastor

**Affiliations:** Instituto de Bioquímica Vegetal y Fotosíntesis, Consejo Superior de Investigaciones Científicas and Universidad de SevillaSevilla, Spain

**Keywords:** regulatory RNA, cyanobacteria, heterocyst, dRNA-Seq, phylogenetic conservation

## Abstract

Small RNAs (sRNAs) are a growing class of non-protein-coding transcripts that participate in the regulation of virtually every aspect of bacterial physiology. Heterocystous cyanobacteria are a group of photosynthetic organisms that exhibit multicellular behavior and developmental alternatives involving specific transcriptomes exclusive of a given physiological condition or even a cell type. In the context of our ongoing effort to understand developmental decisions in these organisms we have undertaken an approach to the global identification of sRNAs. Using differential RNA-Seq we have previously identified transcriptional start sites for the model heterocystous cyanobacterium *Nostoc* sp. PCC 7120. Here we combine this dataset with a prediction of Rho-independent transcriptional terminators and an analysis of phylogenetic conservation of potential sRNAs among 89 available cyanobacterial genomes. In contrast to predictive genome-wide approaches, the use of an experimental dataset comprising all active transcriptional start sites (differential RNA-Seq) facilitates the identification of bona fide sRNAs. The output of our approach is a dataset of predicted potential sRNAs in *Nostoc* sp. PCC 7120, with different degrees of phylogenetic conservation across the 89 cyanobacterial genomes analyzed. Previously described sRNAs appear among the predicted sRNAs, demonstrating the performance of the algorithm. In addition, new predicted sRNAs are now identified that can be involved in regulation of different aspects of cyanobacterial physiology, including adaptation to nitrogen stress, the condition that triggers differentiation of heterocysts (specialized nitrogen-fixing cells). Transcription of several predicted sRNAs that appear exclusively in the genomes of heterocystous cyanobacteria is experimentally verified by Northern blot. Cell-specific transcription of one of these sRNAs, NsiR8 (nitrogen stress-induced RNA 8), in developing heterocysts is also demonstrated.

## Introduction

Small RNAs (sRNAs) are involved in virtually every aspect of bacterial physiology and especially in adaptation to variable nutrient availability and biotic or abiotic stresses (Storz et al., 2011). Regulation of bacterial physiology can no longer be understood without analyzing the participation of sRNAs. Regulatory sRNAs exert posttranscriptional control by a variety of mechanisms, including in many cases the interaction with mRNAs at positions involved in translation initiation, therefore interfering with accumulation of the corresponding proteins (Wagner and Romby, 2015). In recent years, increasing efforts have been made to the identification of the diverse roles of these molecules in all groups of bacteria, from pathogens to environmentally relevant organisms (Wagner and Romby, 2015).

Photosynthetic cyanobacteria, considered the ancestors of chloroplasts, are the most abundant photosynthetic organisms on Earth and therefore crucial to the renewal of atmospheric oxygen and the food chain. Despite being vulnerable to certain stresses such as excess light or UV irradiation, their wide ecological distribution and metabolic versatility suggest the existence of complex regulatory processes leading to adaptation and likely involving sRNAs-controlled mechanisms. In addition, many filamentous cyanobacteria exhibit developmental alternatives, including differentiation of specialized nitrogen-fixing cells, the heterocysts, spore-like cells called akinetes, or motile filaments called hormogonia (Flores and Herrero, 2010). These developmental alternatives involve transcriptional programs that are induced exclusively under specific physiological situations or even in specific cells of the filaments. Differentiation of heterocysts, in which nitrogen fixation is confined, leads to a multicellular behavior in which two different cell types cooperate to achieve growth of the filament as a whole (Muro-Pastor and Hess, 2012). Heterocyst-differentiation, a developmental response to nitrogen deficiency, is ultimately regulated by NtcA, the Crp/Fnr family regulator that operates nitrogen control in cyanobacteria (Herrero et al., 2004), but also under control of HetR, a regulator of cell differentiation (Buikema and Haselkorn, 1991a). Recent data concerning bacterial regulatory circuits highlight the interplay between transcriptional control operated by transcription factors and posttranscriptional control operated by sRNAs (Mandin and Guillier, 2013).

Several examples of sRNA-mediated regulation have recently been described in cyanobacteria, a group of organisms in which posttranscriptional regulation mediated by non-coding RNAs is emerging as a new layer of control of key processes, including photosynthesis (Wilde and Hihara, 2015). Whereas light-regulated PsrR1 (photosynthesis regulatory RNA 1) affects photosynthetic functions (Georg et al., 2014), NtcA-regulated NsiR4 (nitrogen stress induced RNA 4) has recently been demonstrated to participate in posttranscriptional regulation of glutamine synthetase (Klähn et al., 2015). In both cases, the interaction of the sRNA with its target mRNA(s) takes place at positions that include the start codon, therefore interfering with translation. In heterocystous cyanobacteria, HetR-dependent NsiR1 (nitrogen stress induced RNA 1) has been identified as a very early marker of cells undergoing differentiation as heterocysts (Muro-Pastor, 2014) and could be involved in regulation of this developmental process.

The use of advanced methodologies for massive transcriptomic analysis has revealed that primary transcriptomes of bacteria include an unexpectedly high amount of non-coding transcripts (Sorek and Cossart, 2010; Lasa et al., 2012). In the cyanobacterial transcriptomes analyzed so far, including those of *Synechocystis* (Mitschke et al., 2011a; Kopf et al., 2014), *Nodularia spumigena* (Voss et al., 2013), *Trichodesmium erythraeum* (Pfreundt et al., 2014), or heterocyst-forming cyanobacterium *Nostoc* sp. PCC 7120 (Mitschke et al., 2011b), transcription of non-coding sequences accounts for more than 10% of the active promoters.

Global approaches to the identification of sequences possibly encoding sRNAs in cyanobacterial genomes have been carried out by a variety of procedures, including computational prediction followed by experimental validation (Axmann et al., 2005; Voss et al., 2009; Ionescu et al., 2010), microarray-based approaches (Steglich et al., 2008; Georg et al., 2009), and RNA sequencing (Mitschke et al., 2011a,b; Voss et al., 2013; Kopf et al., 2014; Pfreundt et al., 2014). For a recent review see (Kopf and Hess, 2015). Differential RNA-Seq (dRNA-Seq; Sharma and Vogel, 2014), a variant of RNA-Seq that allows precise identification of all active transcription start sites (TSSs) proved as the most powerful approach to the identification of RNAs being actually transcribed. We have previously applied this technique to the analysis of the transcriptome of *Nostoc* (*Anabaena*) sp. PCC 7120, a model heterocyst-forming cyanobacteria, resulting in a genome-wide map of all the positions in which active promoters lead to transcription of different types of RNAs, including antisense and putative sRNAs (Mitschke et al., 2011b). Because nitrogen deprivation leads to dramatic transcriptional changes and ultimately to differentiation of heterocysts, the transcriptional responses to nitrogen step-down were analyzed both in the wild type and in a *hetR* mutant unable to differentiate heterocysts. Two major categories of nitrogen-regulated transcripts, NtcA-dependent and HetR-dependent, were defined, the latter likely involved in differentiation of heterocysts (Mitschke et al., 2011b).

In this work we have undertaken a predictive approach to the identification of sequences transcribed as sRNAs in cyanobacteria, using *Nostoc* sp. PCC 7120 as reference genome. We have combined the positions of active TSSs from intergenic regions and predicted Rho-independent transcriptional terminators. The sequences of all possible intergenic segments flanked by a TSS and a transcriptional terminator are taken as potentially transcribed and further analyzed. Additionally, because conservation of non-coding sequences can be hypothesized to suggest functionality (of a sRNA in this case), we compare all sequences possibly encoding sRNAs in the reference genome with all other genomes available for cyanobacteria with the exception of the fast-evolving clade of *Prochlorococcus* and marine *Synechococcus*. Experimental validation confirms transcription of several previously unknown sRNAs predicted by the algorithm implemented, including NsiR8 (nitrogen stress induced RNA 8), a new HetR-dependent heterocyst-specific sRNA.

## Materials and methods

### Strains and growth conditions

Cultures of wild-type *Nostoc* sp. PCC 7120, *hetR* mutant 216 (Buikema and Haselkorn, 1991b) and *ntcA* mutant CSE2 (Frías et al., 1994) were bubbled with an air/CO_2_ mixture (1% v/v) and grown photoautotrophically at 30°C in BG11_0_C medium (Rippka et al., 1979) lacking NaNO_3_ but containing 6 mM NH_4_Cl, 10 mM NaHCO_3_, and 12 mM N-tris(hydroxymethyl)methyl-2-aminoethanesulfonic acid-NaOH buffer (pH 7.5) until exponential phase. To induce nitrogen deficiency, cells grown in the presence of ammonium were collected by filtration using nitrocellulose membranes (0.45 μm pore diameter), washed with and resuspended in nitrogen-free BG11_0_C medium. RNA samples were isolated from cells taken at the time points indicated after removing combined nitrogen from the media. For the experiment shown in **Figure 4D**, the cells were grown at 30°C in BG11C medium containing NaNO_3_ (Rippka et al., 1979).

### Plasmid construction and fluorescence microscopy

The transcriptional fusion between the promoter region of NsiR8 (PCR-amplified with oligonucleotides NC-7-4 and NC-7-5, see Table S1) and *gfpmut2* was constructed and placed in a neutral site in the alpha megaplasmid of *Nostoc* sp. PCC 7120 as described (Muro-Pastor, 2014). Fluorescence of green fluorescent protein was monitored by collection across a window of 500–538 nm as described (Muro-Pastor, 2014).

### RNA isolation, northern blot, and primer extension analysis

Total RNA was isolated using hot phenol as described (Mohamed and Jansson, 1989) with the following modifications. Hot phenol was added to the cells immediately after addition of the lysis buffer and incubation was carried out at 65°C for 5 min. Further extractions were carried out (once each) with hot phenol, phenol:chloroform (1:1) and chloroform, followed by precipitation of RNA by addition of one volume of isopropanol. Northern blot and primer extension analysis of 5′ ends was performed as previously described (Muro-Pastor et al., 1999, 2002; Steglich et al., 2008). ^32^P-labeled probes for Northern blot were prepared with Taq DNA polymerase using a PCR fragment as template in a reaction with α-^32^P-dCTP and one single oligonucleotide as primer (corresponding to the complementary strand of the sRNA). ^32^P-labeled oligonucleotides (labeled with polynucleotide kinase and γ-^32^P-dATP) were used for primer extension analysis and Northern blot of the RNA transcribed from position 4547237r. Oligonucleotides used for primer extension and to amplify PCR products used as templates to prepare ^32^P-labeled probes are shown in Table S1.

### Prediction of transcriptional terminators

The seven replicons (chromosome and six plasmids) of the reference genome used (*Nostoc* sp. PCC 7120) were downloaded from RefSeq (NCBI Reference Sequence Database). Rho-independent transcriptional terminators were predicted on each replicon using TransTermHP 2.08 with default parameters (Kingsford et al., 2007).

### Homology searches

A local database with 89 cyanobacterial genomes (Table S2) downloaded from NCBI was created using makeblastdb, part of BLAST+ (Camacho et al., 2009). In the case of unfinished genomes, the most advanced assembly of scaffolds was downloaded manually (12/20/2014). Comparisons of sequences selected by the algorithm from the genome of *Nostoc* sp. PCC 7120 with all other genomes was carried out locally using BLASTn with default parameters. Only those hits showing identity over at least 35% of the length of the query were taken as positives. For the definition of RNA families all possible segments transcribed as sRNAs in the reference genome were combined in a local database and each possible sRNA compared to the rest of the sequences in the database.

### Multiple alignments and prediction of secondary structure of sRNAs

Multiple alignments of homologous sequences were obtained using ClustalW 2.0 with default parameters (Larkin et al., 2007). The secondary structure of sRNAs was predicted using RNAalifold (using the multiple alignment previously created by ClustalW as input) or mLocARNA with default parameters (Bernhart et al., 2008; Will et al., 2012).

### Prediction of putative small ORFs

RNAcode (Washietl et al., 2011) was run for every alignment containing more than three sequences. Only putative peptides potentially translated from the same strand as the predicted sRNAs with a *p*-value lower than 0.05 were taken as positives. Web logo (Crooks et al., 2004) was used to analyze the sequence of peptides that could be translated from ncl0160 homologs.

### Script

The pipeline described in this work, including all software involved, was implemented in a single script using Python 3.7, with some Biopython modules (Cock et al., 2009). Details on the script are available upon request. Several parameters can be modified at user's will. The set of results obtained depends on the specific parameters used concerning size of the predicted sRNAs (ranging from 45 to 350 nucleotides in the results presented), length of the homologous segment used as threshold in the search of homologs by BLASTn (35% of the length of the input segment), length of hairpin in the transcriptional terminator, etc. Modifications of these parameters depending on specific needs might produce different outputs therefore reducing/expanding the number of predicted sRNAs.

The final output of 327 predicted sRNAs shown in Table S3 (see Section Results and Discussion) takes into account two additional criteria. When several TSSs are associated with one terminator, only the TSS with the highest number of RNA-Seq reads was included. When several TSS with similar numbers of reads are associated with one terminator but one of the predicted sRNAs had a greater number of detected homologs this TSS was chosen for inclusion in the table.

## Results and discussion

### Genome-wide identification of sequences potentially transcribed as sRNAs in *Nostoc* sp. PCC 7120

We have carried out a prediction of genomic sequences that could encode sRNAs using a TSS dataset from a dRNA-Seq-based transcriptomic analysis of *Nostoc* sp. PCC 7120. Such dataset comprises 13,705 active TSSs that where identified on the basis of more than 50 RNA-Seq reads mapped to the same 5′ end (Mitschke et al., 2011b). In order to extend the prediction also to sRNAs that could be under-represented in RNA-Seq data (see below), we implemented a much lower threshold and therefore used an extended dataset comprising the 48,539 positions for which at least 8 reads were mapped to the same 5′ end. We then selected only those TSS located in intergenic regions (at least 200 nt upstream from the closest annotated gene on the same strand) and therefore likely corresponding to transcription of non-coding RNAs (annotated as nTSS). A prediction for Rho-independent transcriptional terminators in the genome of *Nostoc* sp. PCC 7120 was carried out for each of the seven replicons of this organism (chromosome and six plasmids, obtained from the RefSeq database) rendering a total of 3364 possible terminators.

**Figure 1:**
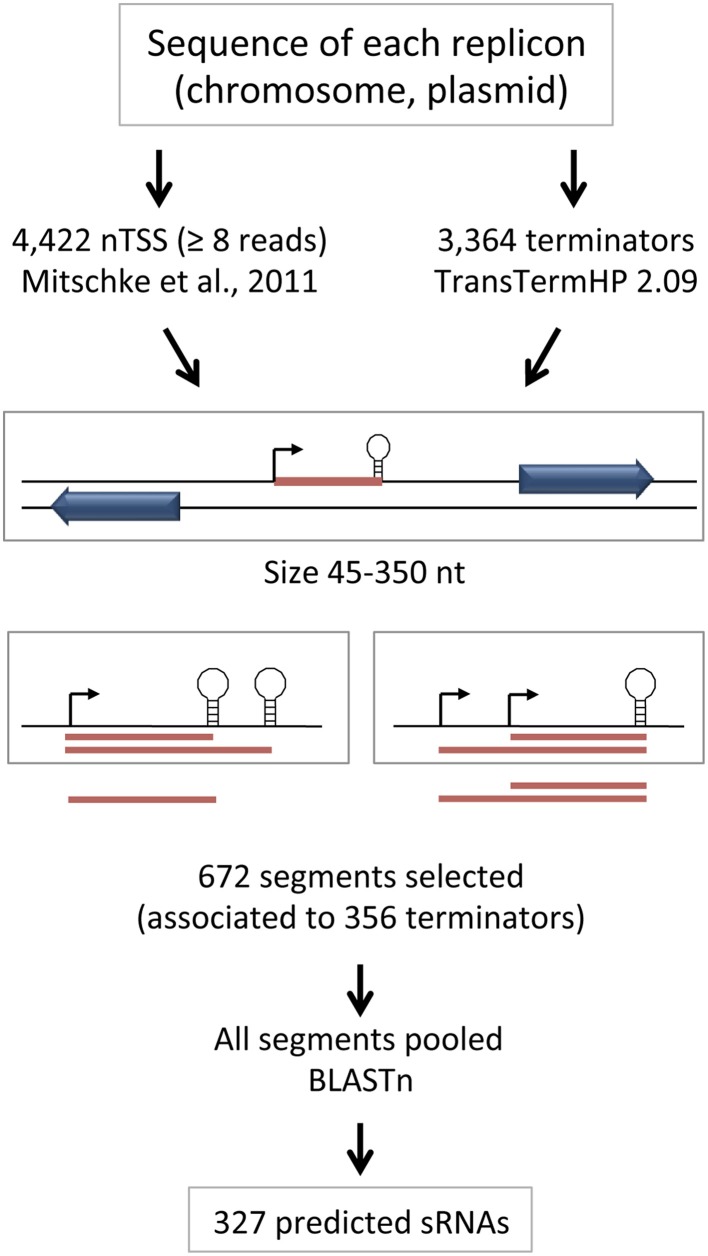
**Outline of the procedure used for prediction of sRNAs**. The approach for initial selection of segments potentially corresponding to sRNAs and the decisions taken in cases where several predicted terminators can be associated to one single TSS or multiple TSS can be associated to one single predicted terminator are shown.

We implemented a pipeline (outlined in Figure 1) that starts by combining all positions corresponding to the 4422 transcriptional start sites annotated as nTSSs and the 3364 predicted Rho-independent transcriptional terminators (see Section Materials and Methods for details). Segments that start at a TSS (from the TSS dataset) and end at a Rho-independent terminator (from the prediction) are taken as potentially transcribed. Such segments, with size limits arbitrarily set between 45 and 350 nucleotides including the predicted terminator hairpin, but not the poly U, were selected for further analysis. In those cases where several terminators could be associated to one single TSS, only the shortest segment was selected, since transcription likely ends at the first terminator encountered by RNA polymerase. In those cases where several TSS could be associated to one terminator, all possible segments were selected by the algorithm. A total of 672 segments, associated to 356 terminators, were therefore selected and grouped.

Upon inspection of the selected sequences we noticed that for some of the predicted sRNAs there were several nearly identical copies in the reference genome that appeared among the sequences selected by the algorithm, indicating that more than one copy was associated to a nTSS defined by RNA-Seq (and therefore being actively transcribed under the growth conditions used). Thus, we pooled all predicted sequences resulting from the algorithm and compared them by BLASTn, so that identical sequences were further grouped into families. Each family is represented by only one member in the output table (usually corresponding to the TSS with the highest number of reads, but see Section Materials and Methods for details), therefore leading to a final number of 327 entries. The complete table containing all results for the seven replicons is presented in Table S3. Each predicted sRNA is identified with the position of the TSS in the corresponding replicon followed by f (forward) or r (reverse) to indicate the strand. For each TSS, the number of RNA-Seq reads (Mitschke et al., 2011b) for the two strains (wild type and *hetR* mutant) and the two nutritional conditions analyzed (growth in the presence of ammonia or in the absence of combined nitrogen for 8 h) is also included.

As a first verification of the performance of the algorithm the results were compared to previously available information on non-coding RNAs of *Nostoc* sp. PCC 7120. Previously described NsiR1 (Ionescu et al., 2010) and NsiR3 (Mitschke et al., 2011b), as well as the homologs of widely conserved cyanobacterial RNAs Yfr1 and Yfr2, first identified in relatively distant, unicellular strains (Axmann et al., 2005; Voss et al., 2007, 2009; Gierga et al., 2009) were among the results of the prediction (See Table S3). Some well characterized RNAs are however not expected to appear in the prediction because they are longer than the fixed size threshold of 350 nucleotides (*rnpB* encoding the RNA component of RNase P and *ssrA* encoding tmRNA) or because their TSS are not annotated as nTSS in the RNA-Seq dataset (*ssaA* encoding 6S RNA and *ffs* encoding 4.5S RNA). The latter is also the case for NsiR4 (Klähn et al., 2015), transcribed in close proximity and tail to tail with *alr3725* from a TSS annotated as antisense TSS. As an additional validation of the results of the algorithm, we analyzed transcription of 14 previously undescribed predicted sRNAs by Northern blot. In all cases transcription of an RNA of the predicted size was confirmed, demonstrating the reliability of the prediction (Table S3, see below).

### Strong conservation of several predicted sRNAs across cyanobacterial lineages

Phylogenetic conservation can provide additional clues concerning possible relevance or function of a putative sRNA and must be evaluated for each particular case. Although lack of conservation does not preclude that one particular sRNA is in fact functional, strong phylogenetic conservation would suggest the existence of selective pressure and therefore functional relevance associated to a conserved sRNA.

We therefore implemented homology searches between each of the 627 segments extracted from the reference genome (*Nostoc* sp. PCC 7120) and other 88 completed or draft genomes from phylogenetically diverse cyanobacteria (Table S2), 26 of which correspond to the clade of heterocystous strains that includes the model organism used as reference. The marine *Prochlorococcus*/*Synechococcus* lineage was excluded because genomes in this group show strong divergence from the rest of cyanobacterial genomes. Because of the wide range of genomes included in the comparison and to reduce false positives, in addition to applying the default *e*-value of BLASTn, only segments with a minimum length of homology corresponding to 35% of the input sequence were taken as positives in the automatic classification of results. Two columns in the output table (Table S3) include the counts of homologs for each predicted sRNA in all 89 genomes or specifically in the 27 genomes of heterocystous cyanobacteria. The alignments generated by the algorithm for each predicted sRNA and its homologs in other cyanobacterial genomes were visually inspected, manually curated and used as input for RNAcode (Washietl et al., 2011), a software that predicts protein coding regions in a set of homologous nucleotide sequences. Several sRNAs in the output of the algorithm were predicted to encode small proteins (annotated in Table S3).

**Figure 2:**
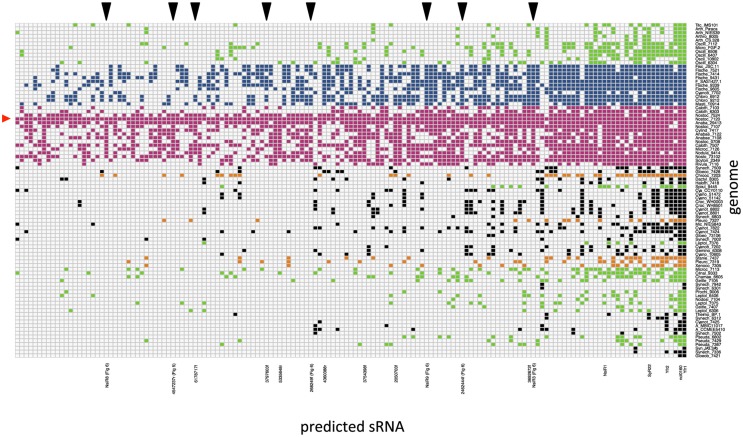
**Graphic map of the presence of homologs to predicted sRNAs from ***Nostoc*** sp. PCC 7120 in cyanobacterial genomes**. Only the 151 predicted sRNAs with at least 10 homologs are included. Genomes are arranged and color-coded as in Table S2 (Shih et al., 2013) as follows: filamentous non-heterocystous in green, heterocystous ramified in blue, heterocystous non-ramified in pink, baeocystous in orange, and unicellular in black. sRNAs are arranged according to phylogenetic conservation (sRNAs with highest number of homologs appear in the right side of the map). The positions corresponding to previously described sRNAs, or new sRNAs experimentally validated in this work are labeled under the map. The reference organism is marked with a red triangle on the left side. The position of several validated sRNAs with homologs identified only in the genomes of heterocystous cyanobacteria is indicated with black triangles above the map.

The presence of homologs to the possible sRNAs predicted from the reference genome in all other 88 genomes is presented in a graphic map (Figure 2). Only the 151 predicted sRNAs with homologs in more than 10 genomes are included for clarity. Note that, as expected from the fact that the reference genome used for the prediction corresponds to a heterocystous strain, homologs to the predicted sRNAs are more abundant among heterocystous strains (represented in pink and blue).

**Figure 3:**
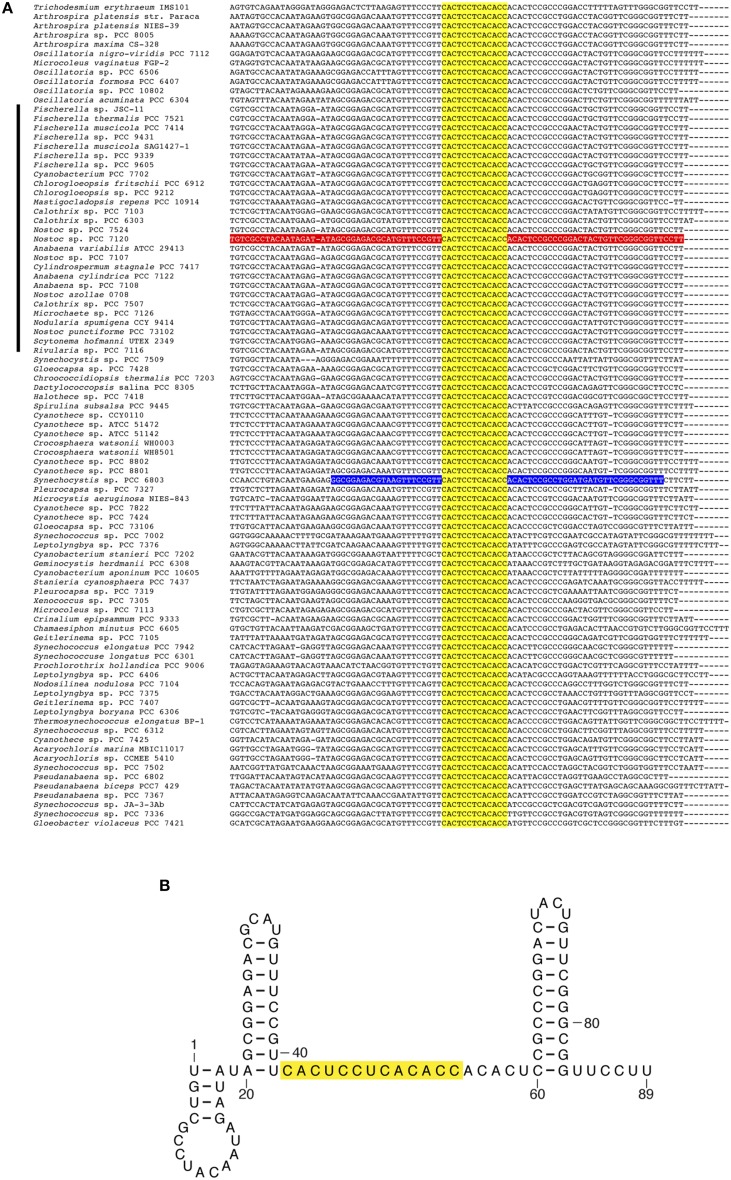
**Alignment of Yfr1 homologs and predicted secondary structure of Yfr1 from ***Nostoc*** sp. PCC 7120. (A)** Alignment of sequences encoding Yfr1 homologs in 89 cyanobacterial genomes. The predicted transcript in *Nostoc* sp. PCC 7120 is shown in red. The described Yfr1 for *Synechocystis* sp. PCC 6803 (Mitschke et al., 2011a) is shown in blue. Genomes of heterocystous cyanobacteria are indicated with a black bar on the left side. **(B)** Predicted secondary structure of Yfr1 from *Nostoc* sp. PCC 7120. The sequence of a fully conserved motif is shown in yellow.

Sequences encoding several predicted sRNAs appear very strongly conserved across the whole phylum. For instance, homologs to previously described Yfr1 (cyanobacterial functional RNA1) appear in every cyanobacterial genome analyzed (Figure 3). Yfr1, initially described (Axmann et al., 2005) as a 54-57-nt RNA in the marine *Prochlorococcus*/*Synechococcus* lineage was later identified in several cyanobacterial lineages (Voss et al., 2007), including four heterocystous strains. Our approach identifies the Yfr1 homolog as a transcribed sRNA in *Nostoc* sp. PCC 7120 (TSS 56433f in Table S3). Similarly, homologs of Yfr2 (cyanobacterial functional RNA 2) (Axmann et al., 2005) and ncl0160 (Mitschke et al., 2011a), previously identified in *Synechocystis* sp. PCC 6803, are widely distributed in both heterocystous and non-heterocystous strains according to the homology searches implemented in our pipeline.

Careful inspection of some of the alignments for sRNAs with many homologs allows the identification of conserved features. For instance, conservation of the sequence 5′CACUCCUCACACC3′ in all the homologs of Yfr1 (Figure 3), points to this presumably unpaired region as critical for Yfr1 function, as previously suggested on the basis of a smaller number of homologs (Voss et al., 2007). Similarly, the alignment of all homologs of ncl0160 (including those for the 27 heterocystous strains) reveals a strongly conserved region that could be translated as a conserved small 23–25 aa peptide (Figure S1). The predicted start codon for this small peptide (mostly ATG, but also GTG in a few cases) is always preceded by sequences potentially conforming a ribosome binding site. All three possible stop codons are observed among the 83 homologs identified (47 occurrences of TAA, 34 of TAG, and 2 of TGA). Therefore, ncl0160 is likely translated as a small peptide. The predicted secondary structure of the ncl0160 homolog in *Nostoc* sp. PCC 7120 and a weblogo obtained for the putatively translated peptide in 83 strains are shown in Figure S2. The two above described examples, Yfr1 and ncl0160, show that the identification of conserved sequences in sRNA homologs that are present in a large number of cyanobacterial genomes might help in the identification of relevant features.

### Phylogenetically conserved sRNAs represented by low numbers of reads in the RNA-seq dataset

**Figure 4:**
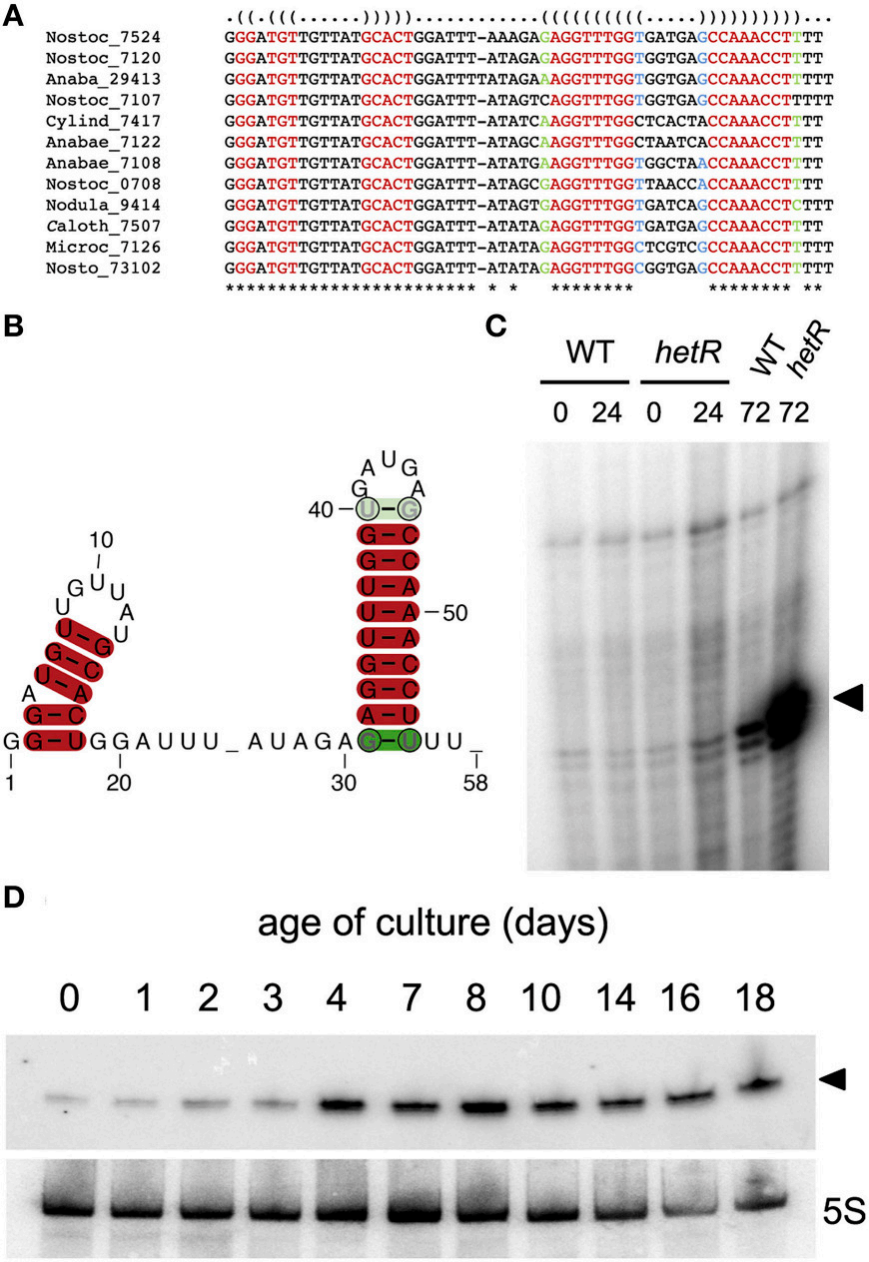
**Predicted sRNA transcribed from position 4547237r in *Nostoc* sp. PCC 7120. (A)** Alignment of homologs found in the genomes of heterocystous cyanobacteria. The identifiers for the genomes are described in Table S2. **(B)** Consensus secondary structure prediction by RNAalifold (Bernhart et al., 2008). **(C)** Primer extension of RNA samples from the wild type *Nostoc* sp. PCC7120 (WT) and the *hetR* mutant subjected to combined nitrogen deficiency for the number of hours indicated. Extension products corresponding to position 4547237r are indicated with a black triangle. **(D)** Northern blot showing increased expression of the predicted sRNA (indicated with a black triangle) with density of the cultures. Lower panel shows hybridization to a 5S probe.

The approach we described here is based on a dataset of transcriptional start sites defined by dRNA-Seq. The number of reads associated to each TSS by RNA-Seq is influenced by several factors, including size of the transcript (transcripts with small sizes are under-represented) or transcription under specific physiological conditions different from the standard growth conditions (nutrient limitation, stationary phase, changes in light intensity, etc). Also, sRNAs that are processed in the 5′ end are under-represented because the dRNA-Seq procedure counter-selects processed, non-primary 5′ ends. In this context we hypothesized that the analysis of phylogenetic conservation of possibly transcribed sequences could facilitate the identification of true sRNAs with small numbers of reads. As an example, among the phylogenetically conserved predicted sRNAs (with homologs in more than 10 heterocystous strains), we have experimentally verified transcription of one of the predicted sRNAs with the lowest number of reads (Figure 4).

According to our predictive approach, transcription from position 4547237r, for which only eight reads are mapped in dRNA-Seq, would produce an sRNA slightly above 50 nucleotides in size, therefore in the lower size limit for the fractionated RNA sample used in RNA-Seq. 12 out of the 27 genomes for heterocystous strains included in the homology search contain sequences that the algorithm identifies as homologs with the parameters used (Figure 4A). The consensus secondary structure is shown in Figure 4B. We verified transcription of this potential sRNA by primer extension and Northern blot. A small sRNA of the expected size was in fact transcribed from position 4547237r in cells growing under different physiological situations, including a long period of nitrogen deficiency (Figure 4C), or advanced stages of growth in the presence of combined nitrogen (Figure 4D). Therefore, even with only eight RNA-Seq reads associated to TSS 4547237r, transcription of the corresponding sRNA could be readily detected by Northern blot, confirming the accuracy of the predictive algorithm. This sRNA seems to be induced under stressful situations such as prolonged nitrogen deficiency or stationary phase but, interestingly, homologs are exclusively found in the genomes of heterocystous strains (see below).

### sRNAs in heterocystous cyanobacteria

Heterocystous cyanobacteria, represented by 27 genomes in our study, constitute a phylogenetically coherent group of strains that share the ability to differentiate heterocysts, a specialized cell type devoted to fixation of N_2_. The TSS dataset for the model heterocystous cyanobacterium *Nostoc* sp. PCC 7120 contains combined information for the transcriptome of the wild-type strain and a *hetR* mutant (heterocyst-defective) both in the presence of combined nitrogen and after 8 h of nitrogen deficiency (Mitschke et al., 2011b). Because we are interested in the identification of regulatory sRNAs that might be involved in adaptation to changes in nitrogen supply and specifically in the differentiation of heterocysts, we have further analyzed predicted sRNAs that seem exclusive of this group of bacteria.

**Figure 5:**
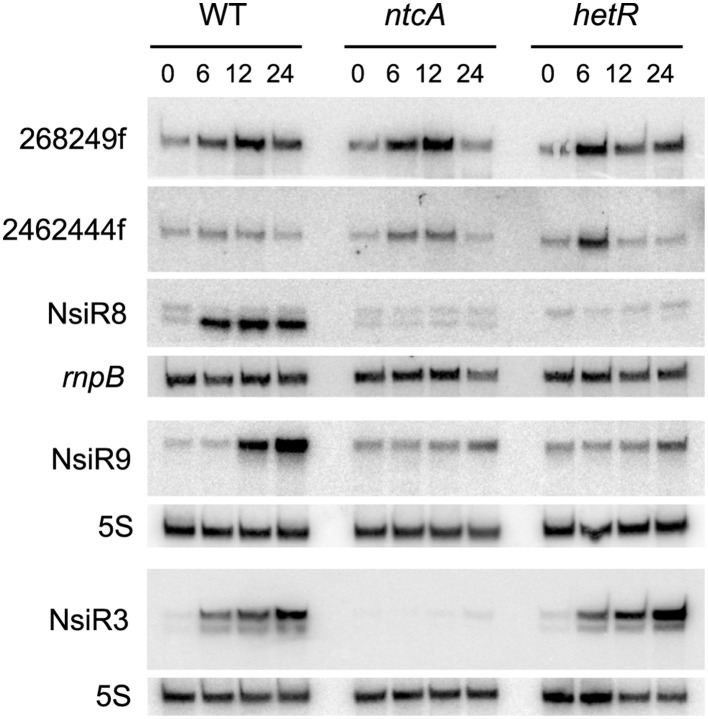
**Expression of several predicted sRNAs in ***Nostoc*** sp. PCC 7120**. RNA samples were isolated from cells of the wild type (WT) or *ntcA* and *hetR* mutants subjected to nitrogen deficiency for the number of hours indicated and subjected to Northern blot with probes corresponding to sRNAs 268249f, 2462444f, and NsiR8 (membrane 1), NsiR9 (membrane 2), and NsiR3, included for comparison (membrane 3). *rnpB* (membrane 1) or 5S RNA (membranes 2 and 3) were used as loading and transfer controls.

Table 1 contains information on selected predicted sRNAs with homologs found exclusively in heterocystous cyanobacteria. RNA-Seq data (Mitschke et al., 2011b) indicate that some of them are strongly transcribed and also appear regulated by the availability of combined nitrogen. Homologs of predicted sRNAs starting at positions 268249f (608,990 total reads in RNA-Seq) and 2462444f (151,091 total reads in RNA-Seq) would be encoded in 18 and 24 of the 27 genomes for heterocystous cyanobacteria, respectively, according to the automatic homology search implemented in the pipeline. Northern blot confirmed that both predicted sRNAs were actually transcribed as transcripts of the expected size, according to the predicted transcriptional terminators (340 and 258 nucleotides, respectively; Figure 5). In agreement with RNA-Seq data, expression of both sRNAs was induced in the wild type strain under nitrogen limitation, but the induction also took place in *ntcA* or *hetR* mutants, indicating that the transcriptional regulation of these sRNAs is independent of the two major regulators of the response to nitrogen deficiency (NtcA) and differentiation of heterocysts (HetR). Therefore, we expect these two sRNAs to be involved in the response to other stresses as well.

**Table 1:** **Selected^a^ predicted sRNAs from ***Nostoc*** sp. PCC 7120 with homologs in some of the 27 genomes of heterocyst forming cyanobacteria**.

			**Reads RNA-Seq (Mitschke et al., 2011b)**				
			**Wild type**		***hetR***		
**Position**	**Homologs^b^**	**Length**	**${\text{NH}}_{4}^{+}$**	**8 h N_2_**	**${\text{NH}}_{4}^{+}$**	**8 h N_2_**	**Comment**
5452083f	27	99	7	449	8	412	NsiR3 (Mitschke et al., 2011b); Figure 5
6274486f	26	169	39	44	60	20	
2462444f	24	258	15795	77486	7505	50305	Verified by Northern **(**Figure 5**)**
39081f	23	246	7	18	14	9	
4261534f	23	344	313	391	480	144	
2375634r	22	116	8	43	7	19	
4451847f	22	122	8	138	9	11	NsiR9; Verified by Northern **(**Figure 5**)**
5245514r	22	120	174	135	94	79	
4499448r	21	164	25	28	48	16	
4408600f	19	236	1	71			
19177f	18	62	1		8		
268249f	18	340	36892	415651	16043	140404	Verified by Northern **(**Figure 5**)**
1099171f	17	228	5	15	11	18	
4772591r	17	155	92	60	69	37	
1938093f	15	70	4	5	5	5	
3797603f	15	191	808	476	10462	6648	Verified by Northern (not shown)
3994161f	15	222	5	1	1	1	
4040345r	15	89	40	14	24	15	
5439722r	15	92	2	3	1	4	
1171556r	13	119	6	4	1	3	
3514677r	13	95	1		7	3	
4434324f	13	232	9	8	1	5	
1748547f	12	194	6	14	1	20	
1985886f	12	126	10	4	1	1	
3376483f	12	219	91	91	138	81	
3807040r	12	349	1	10		5	
4547237r	12	51	4	1	1	2	Verified by Northern **(**Figure 4**)**
4837354f	12	112	2	9	19	2	
535664r	12	135	25	16	17	15	
6178717f	12	217	1783	1447	179817	4478	Verified by Northern (not shown)
2066070r	11	128	1			10	
3756266r	11	326	1			9	
4547556r	11	171	115	1708	26	8	NsiR8; Verified by Northern **(**Figure 5**)**
4895315r	11	140	15	39	20	20	
6342036f	11	153	2	2	1	4	

a *Predicted sRNAs with homologs found exclusively in genomes from heterocyst forming cyanobacteria. Only chromosomally-encoded sRNAs with more than 10 putative homologs are included in the table*.

b *Number of homologs identified in the 27 genomes of heterocystous cyanobacteria included in this study. Lines corresponding to selected sRNAs whose transcription in Nostoc sp. PCC 7120 has been verified by Northern blot are shaded in gray*.

In addition to previously described NsiR3 (Mitschke et al., 2011b), several other predicted sRNAs would be transcribed from N-regulated TSS and were therefore named nitrogen stress induced RNAs. Northern blot showed that transcripts with sizes that are in good agreement with those predicted by the algorithm were observed for 4547556r (171 nt), named NsiR8, and 4451847f (122 nt), named NsiR9. Consistent with dRNA-Seq data included in Table 1 (Mitschke et al., 2011b), whereas the induction of expression of NsiR3 (included for comparison) required NtcA but not HetR, expression of NsiR8 or NsiR9 (Figure 5) was not induced in a *hetR* mutant, therefore suggesting the last two sRNAs could be related to heterocysts differentiation or function. An schematic representation of the genomic context of the sRNAs included in Figure 5 is shown in Figure S3 using Artemis (Rutherford et al., 2000).

### Transcription of NsiR8 is heterocyst-specific

**Figure 6:**
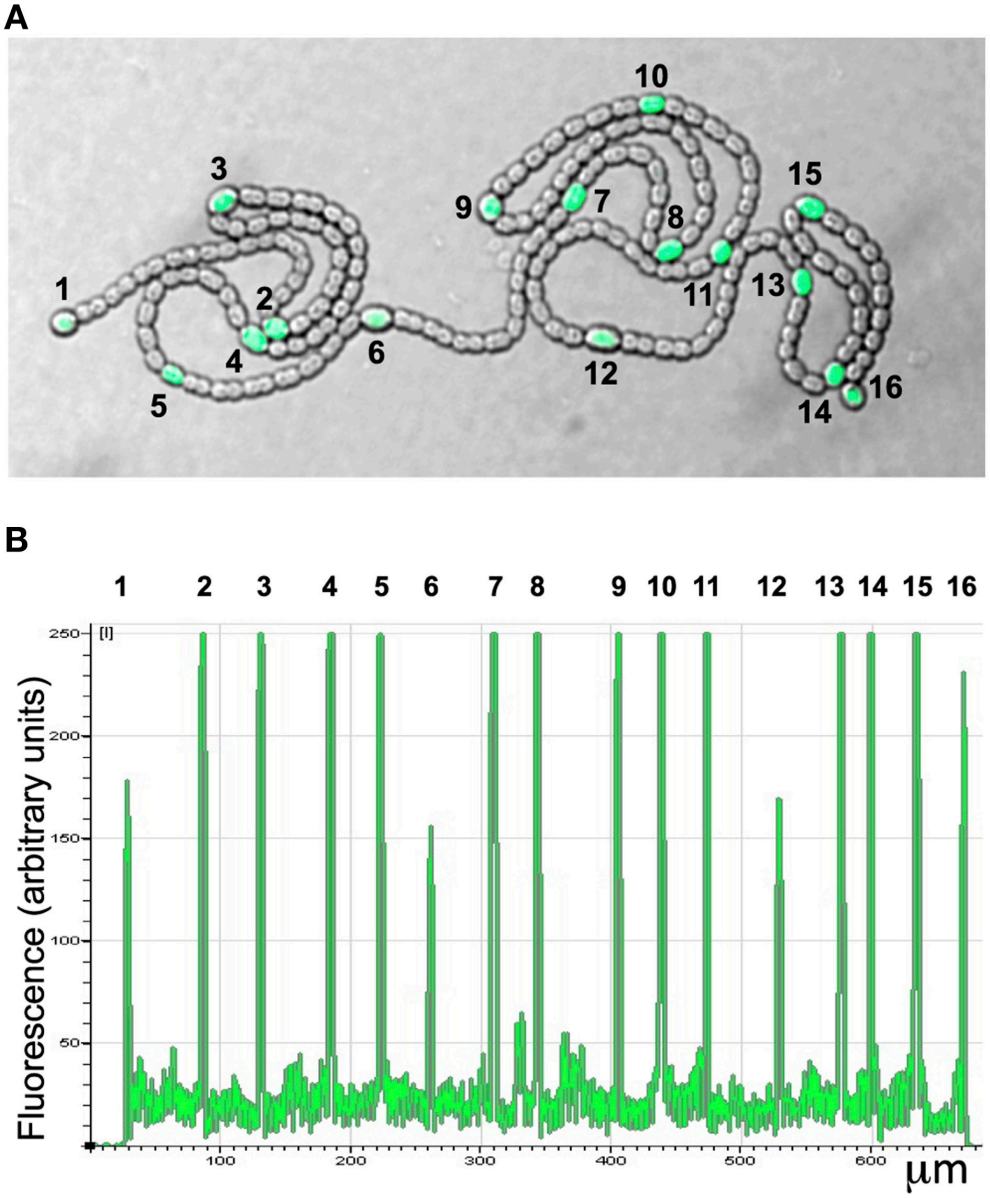
**Expression of P_***nsiR8***_-***gfp*** in nitrogen-fixing filaments of ***Nostoc*** sp. PCC 7120**. **(A)** Confocal fluorescence image of a filament growing on top of nitrogen-free medium. The green (green fluorescent protein) and bright field channels are merged. **(B)** Quantification of the green signal along the filament shown in **(A)**. The position of heterocysts is indicated with numbers.

Because transcription of NsiR8 and NsiR9 required HetR, we hypothesized that these sRNA could be related to the differentiation of heterocysts and, similar to HetR-dependent NsiR1 (Ionescu et al., 2010), perhaps expressed in cells undergoing differentiation (Muro-Pastor, 2014). The promoter of NsiR8 was fused to the *gfpmut2* gene encoding a version of green fluorescent protein and the construct was introduced in *Nostoc* sp. PCC 7120. Expression of GFP under control of the promoter of NsiR8 was analyzed by confocal fluorescence microscopy as previously described (Muro-Pastor, 2014) and found to take place specifically in developing heterocysts (Figure 6).

## Concluding remarks

Differential RNA-Seq-based datasets containing genome-wide maps of active promoters provide unprecedented details on transcription in bacteria. Using a previously available dataset for the model organism *Nostoc* sp. PCC 7120, we have implemented a pipeline for global prediction of sequences potentially encoding sRNAs in cyanobacterial genomes, with a special focus on heterocystous cyanobacteria. The procedure we have implemented could be applied to any group of bacteria for which a dRNA-Seq approach has been undertaken. Although published dRNA-Seq datasets are somewhat variable in their depth and coverage, the pipeline described here does not require a large number of reads mapped for any given TSS. The requirement for the presence of a transcriptional terminator provides further strength to the prediction of a transcribed sequence as a potential sRNA-encoding gene. In fact, transcription of most sRNAs involved in adaptation to certain physiological situations takes place only under those specific circumstances, and therefore their TSSs are under-represented in RNA-Seq datasets obtained under standard culture conditions. Using the predicted sRNA transcribed from position 4547237r, we have demonstrated that, even in the case of a TSS with very low numbers of reads (because of low transcription or small size), the algorithm identifies a transcribed sRNA.

Phylogenetic conservation of sequences predicted to be transcribed as sRNAs in related (or even distant) genomes provides additional information concerning a possible functional role. In the case of *Nostoc* sp. PCC 7120, whereas some of the sRNAs predicted by the algorithm appear widely distributed among relatively distant cyanobacterial strains (e.g., Yfr1, Yfr2), homologs to a few predicted sRNAs are exclusively found in the genomes of heterocystous strains (closely related to the reference genome and therefore sharing common specific physiological traits). For some of the predicted sRNAs, in particular those that are specifically transcribed in heterocysts (e.g., NsiR8) a possible function related to heterocyst differentiation or function can be suspected. Finally, phylogenetic conservation of certain sequences in the predicted sRNAs can suggest translation of a peptide (e.g., in the case of ncl0160 homologs) or possible interactions with other RNA or protein molecules (e.g., in the case of the fully conserved, presumably single-stranded motif in the homologs of Yfr1). The predicted sRNAs described in this work, several of them experimentally validated here, constitute a set of potentially regulatory elements to be analyzed in further detail in heterocystous cyanobacteria.

## Author contributions

AM and MB designed the computational approach. MB wrote scripts and implemented the sRNAs prediction. MB and AV performed all other computational tasks. MB, EO, and AM performed experimental validation of sRNAs. AM prepared *gfp* fusions and performed confocal fluorescence microscopy analysis. All authors were involved in analyzing the results and writing the manuscript.

## Funding

This work was supported by grant BFU2013-48282-C2-1 (from the Spanish Government, Ministerio de Economía y Competitividad, co-financed by European Regional Development Fund) to AM. MB is the recipient of a fellowship from Agencia Estatal CSIC, Spain (Jae Intro) and a contract from Ministerio de Educación Cultura y Deporte, Spain (FPU014/05123).

### Conflict of interest statement

The authors declare that the research was conducted in the absence of any commercial or financial relationships that could be construed as a potential conflict of interest.

## References

[B1] Axmann I. M. Kensche P. Vogel J. Kohl S. Herzel H. Hess W. R. (2005). Identification of cyanobacterial non-coding RNAs by comparative genome analysis. Genome Biol. 6, R73. 10.1186/gb-2005-6-9-r73 16168080 PMC1242208

[B2] Bernhart S. H. Hofacker I. L. Will S. Gruber A. R. Stadler P. F. (2008). RNAalifold: improved consensus structure prediction for RNA alignments. BMC Bioinformatics 9:474. 10.1186/1471-2105-9-474 19014431 PMC2621365

[B3] Buikema W. J. Haselkorn R. (1991a). Characterization of a gene controlling heterocyst differentiation in the cyanobacterium *Anabaena* 7120. Genes Dev. 5, 321–330. 10.1101/gad.5.2.321 1840555

[B4] Buikema W. J. Haselkorn R. (1991b). Isolation and complementation of nitrogen fixation mutants of the cyanobacterium *Anabaena* sp. strain PCC 7120. J. Bacteriol. 173, 1879–1885. 1900504 10.1128/jb.173.6.1879-1885.1991 PMC207717

[B5] Camacho C. Coulouris G. Avagyan V. Ma N. Papadopoulos J. Bealer K.. (2009). BLAST+: architecture and applications. BMC Bioinformatics 10:421. 10.1186/1471-2105-10-421 20003500 PMC2803857

[B6] Cock P. J. Antao T. Chang J. T. Chapman B. A. Cox C. J. Dalke A.. (2009). Biopython: freely available Python tools for computational molecular biology and bioinformatics. Bioinformatics 25, 1422–1423. 10.1093/bioinformatics/btp163 19304878 PMC2682512

[B7] Crooks G. E. Hon G. Chandonia J. M. Brenner S. E. (2004). WebLogo: a sequence logo generator. Genome Res. 14, 1188–1190. 10.1101/gr.849004 15173120 PMC419797

[B8] Flores E. Herrero A. (2010). Compartmentalized function through cell differentiation in filamentous cyanobacteria. Nat. Rev. Microbiol. 8, 39–50. 10.1038/nrmicro2242 19966815

[B9] Frías J. E. Flores E. Herrero A. (1994). Requirement of the regulatory protein NtcA for the expression of nitrogen assimilation and heterocyst development genes in the cyanobacterium *Anabaena* sp. PCC 7120. Mol. Microbiol. 14, 823–832. 10.1111/j.1365-2958.1994.tb01318.x 7534371

[B10] Georg J. Dienst D. Schurgers N. Wallner T. Kopp D. Stazic D.. (2014). The small regulatory RNA SyR1/PsrR1 controls photosynthetic functions in cyanobacteria. Plant Cell 26, 3661–3679. 10.1105/tpc.114.129767 25248550 PMC4213160

[B11] Georg J. Voss B. Scholz I. Mitschke J. Wilde A. Hess W. R. (2009). Evidence for a major role of antisense RNAs in cyanobacterial gene regulation. Mol. Syst. Biol. 5, 305. 10.1038/msb.2009.63 19756044 PMC2758717

[B12] Gierga G. Voss B. Hess W. R. (2009). The Yfr2 ncRNA family, a group of abundant RNA molecules widely conserved in cyanobacteria. RNA Biol. 6, 222–227. 10.4161/rna.6.3.8921 19502815

[B13] Herrero A. Muro-Pastor A. M. Valladares A. Flores E. (2004). Cellular differentiation and the NtcA transcription factor in filamentous cyanobacteria. FEMS Microbiol. Rev. 28, 469–487. 10.1016/j.femsre.2004.04.003 15374662

[B14] Ionescu D. Voss B. Oren A. Hess W. R. Muro-Pastor A. M. (2010). Heterocyst-specific transcription of NsiR1, a non-coding RNA encoded in a tandem array of direct repeats in cyanobacteria. J. Mol. Biol. 398, 177–188. 10.1016/j.jmb.2010.03.010 20227418

[B15] Kingsford C. L. Ayanbule K. Salzberg S. L. (2007). Rapid, accurate, computational discovery of Rho-independent transcription terminators illuminates their relationship to DNA uptake. Genome Biol. 8, R22. 10.1186/gb-2007-8-2-r22 17313685 PMC1852404

[B16] Klähn S. Schaal C. Georg J. Baumgartner D. Knippen G. Hagemann M.. (2015). The sRNA NsiR4 is involved in nitrogen assimilation control in cyanobacteria by targeting glutamine synthetase inactivating factor IF7. Proc. Natl. Acad. Sci. U.S.A. 112, E6243–E6252. 10.1073/pnas.1508412112 26494284 PMC4653137

[B17] Kopf M. Hess W. R. (2015). Regulatory RNAs in photosynthetic cyanobacteria. FEMS Microbiol. Rev. 39, 301–315. 10.1093/femsre/fuv017 25934122 PMC6596454

[B18] Kopf M. Klähn S. Scholz I. Matthiessen J. K. Hess W. R. Voss B. (2014). Comparative analysis of the primary transcriptome of *Synechocystis* sp. PCC 6803. DNA Res 21, 527–539. 10.1093/dnares/dsu018 24935866 PMC4195498

[B19] Larkin M. A. Blackshields G. Brown N. P. Chenna R. McGettigan P. A. McWilliam H.. (2007). Clustal W and clustal X version 2.0. Bioinformatics 23, 2947–2948. 10.1093/bioinformatics/btm404 17846036

[B20] Lasa I. Toledo-Arana A. Gingeras T. R. (2012). An effort to make sense of antisense transcription in bacteria. RNA Biol. 9, 1039–1044. 10.4161/rna.21167 22858676 PMC3551857

[B21] Mandin P. Guillier M. (2013). Expanding control in bacteria: interplay between small RNAs and transcriptional regulators to control gene expression. Curr. Opin. Microbiol. 16, 125–132. 10.1016/j.mib.2012.12.005 23415757

[B22] Mitschke J. Georg J. Scholz I. Sharma C. M. Dienst D. Bantscheff J.. (2011a). An experimentally anchored map of transcriptional start sites in the model cyanobacterium *Synechocystis* sp. PCC6803. Proc. Natl. Acad. Sci. U.S.A 108, 2124–2129. 10.1073/pnas.1015154108 21245330 PMC3033270

[B23] Mitschke J. Vioque A. Haas F. Hess W. R. Muro-Pastor A. M. (2011b). Dynamics of transcriptional start site selection during nitrogen stress-induced cell differentiation in *Anabaena* sp. PCC7120. Proc. Natl. Acad. Sci. U.S.A. 108, 20130–20135. 10.1073/pnas.1112724108 22135468 PMC3250118

[B24] Mohamed A. Jansson C. (1989). Influence of light on accumulation of photosynthesis-specific transcripts in the cyanobacterium *Synechocystis* 6803. Plant Mol. Biol. 13, 693–700. 10.1007/BF00016024 2518835

[B25] Muro-Pastor A. M. (2014). The heterocyst-specific NsiR1 small RNA is an early marker of cell differentiation in cyanobacterial filaments. MBio 5, e01079–e01014. 10.1128/mBio.01079-14 24825011 PMC4030482

[B26] Muro-Pastor A. M. Hess W. R. (2012). Heterocyst differentiation: from single mutants to global approaches. Trends Microbiol. 20, 548–557. 10.1016/j.tim.2012.07.005 22898147

[B27] Muro-Pastor A. M. Valladares A. Flores E. Herrero A. (1999). The *hetC* gene is a direct target of the NtcA transcriptional regulator in cyanobacterial heterocyst development. J. Bacteriol. 181, 6664–6669. 10542167 10.1128/jb.181.21.6664-6669.1999 PMC94130

[B28] Muro-Pastor A. M. Valladares A. Flores E. Herrero A. (2002). Mutual dependence of the expression of the cell differentiation regulatory protein HetR and the global nitrogen regulator NtcA during heterocyst development. Mol. Microbiol. 44, 1377–1385. 10.1046/j.1365-2958.2002.02970.x 12068814

[B29] Pfreundt U. Kopf M. Belkin N. Berman-Frank I. Hess W. R. (2014). The primary transcriptome of the marine diazotroph *Trichodesmium erythraeum* IMS101. Sci. Rep. 4, 6187. 10.1038/srep06187 25155278 PMC4143802

[B30] Rippka R. Deruelles J. Waterbury J. B. Herdman M. Stanier R. Y. (1979). Generic assignments, strain stories and properties of pure cultures of cyanobacteria. J. Gen. Microbiol. 111, 1–61.

[B31] Rutherford K. Parkhill J. Crook J. Horsnell T. Rice P. Rajandream M. A.. (2000). Artemis: sequence visualization and annotation. Bioinformatics 16, 944–945. 10.1093/bioinformatics/16.10.944 11120685

[B32] Sharma C. M. Vogel J. (2014). Differential RNA-seq: the approach behind and the biological insight gained. Curr. Opin. Microbiol. 19, 97–105. 10.1016/j.mib.2014.06.010 25024085

[B33] Shih P. M. Wu D. Latifi A. Axen S. D. Fewer D. P. Talla E.. (2013). Improving the coverage of the cyanobacterial phylum using diversity-driven genome sequencing. Proc. Natl. Acad. Sci. U.S.A. 110, 1053–1058. 10.1073/pnas.1217107110 23277585 PMC3549136

[B34] Sorek R. Cossart P. (2010). Prokaryotic transcriptomics: a new view on regulation, physiology and pathogenicity. Nat. Rev. Genet. 11, 9–16. 10.1038/nrg2695 19935729

[B35] Steglich C. Futschik M. E. Lindell D. Voss B. Chisholm S. W. Hess W. R. (2008). The challenge of regulation in a minimal photoautotroph: non-coding RNAs in *Prochlorococcus*. PLoS Genet. 4:e1000173. 10.1371/annotation/411b74ae-c4ce-43c9-bdd2-60c2bf60e672 18769676 PMC2518516

[B36] Storz G. Vogel J. Wassarman K. M. (2011). Regulation by small RNAs in bacteria: expanding frontiers. Mol. Cell. 43, 880–891. 10.1016/j.molcel.2011.08.022 21925377 PMC3176440

[B37] Voss B. Bolhuis H. Fewer D. P. Kopf M. Moke F. Haas F.. (2013). Insights into the physiology and ecology of the brackish-water-adapted cyanobacterium *Nodularia spumigena* CCY9414 based on a genome-transcriptome analysis. PLoS ONE 8:e60224. 10.1371/journal.pone.0060224 23555932 PMC3610870

[B38] Voss B. Georg J. Schön V. Ude S. Hess W. R. (2009). Biocomputational prediction of non-coding RNAs in model cyanobacteria. BMC Genomics 10:123. 10.1186/1471-2164-10-123 19309518 PMC2662882

[B39] Voss B. Gierga G. Axmann I. M. Hess W. R. (2007). A motif-based search in bacterial genomes identifies the ortholog of the small RNA Yfr1 in all lineages of cyanobacteria. BMC Genomics 8:375. 10.1186/1471-2164-8-375 17941988 PMC2190773

[B40] Wagner E. G. Romby P. (2015). Small RNAs in bacteria and archaea: who they are, what they do, and how they do it. Adv. Genet. 90, 133–208. 10.1016/bs.adgen.2015.05.001 26296935

[B41] Washietl S. Findeiss S. Muller S. A. Kalkhof S. von Bergen M. Hofacker I. L.. (2011). RNAcode: robust discrimination of coding and noncoding regions in comparative sequence data. RNA 17, 578–594. 10.1261/rna.2536111 21357752 PMC3062170

[B42] Wilde A. Hihara Y. (2015). Transcriptional and posttranscriptional regulation of cyanobacterial photosynthesis. Biochim Biophys Acta. 10.1016/j.bbabio.2015.11.002 26549130

[B43] Will S. Joshi T. Hofacker I. L. Stadler P. F. Backofen R. (2012). LocARNA-P: accurate boundary prediction and improved detection of structural RNAs. RNA 18, 900–914. 10.1261/rna.029041.111 22450757 PMC3334699

