# Supplementary material for: Identification of Conserved and Potentially Regulatory Small RNAs in Heterocystous Cyanobacteria
Source: Front Microbiol. 2016 Feb 1;7:48. doi: 10.3389/fmicb.2016.00048 (PMC4734099; doi:10.3389/fmicb.2016.00048)
Supplement: Supplementary file 1 [file Table1.DOCX]

**Table S1. Oligonucleotides used in this work**

| **Oligonucleotide** | **Sequence (5' to 3')** | **Use** |
| --- | --- | --- |
| NC-7-4 | TATTGGATCGATTGATTACAGGTGTAG | NsiR8 promoter for *gfp* fusion |
| NC-7-5 | AATCATCTCGAGCCTCACAATTCCC |  |
| TFM2_2470310-1 | GGATGGGGATTTGTGAGCTA | Probe for Northern (2470310f, homolog of SyR22) |
| TFM2_2470310-2 | TGGAATTAACCAGCCAAACC |  |
| TFM3_3692672-1 | TTAGGGACTGGTGAGCCAAC | Probe for Northern (3692672f) |
| TFM3_3692672-2 | ATCGGCATGACGCTATTTAC |  |
| TFM4_3754398-1 | TAAAGAAATCCATGAACAGC | Probe for Northern (3754398f) |
| TFM4_3754398-2 | TCTAGTCAACAGTCAACAG |  |
| TFM5_4360388-1 | CCACTGCCATCATCTTACC | Probe for Northern (4360388r) |
| TFM5_4360388-2 | GTTCTTGCTTTGAATGTAG |  |
| TFM6_4451847-1 | CCCATTACCTCCCTTGGAGT | Probe for Northern (4451847f, NsiR9) |
| TFM6_4451847-2 | AAAATAGATGCCCGTCATGC |  |
| TFM7_5335648-1 | GTCGAGCCAAGCCTTTATGT | Probe for Northern (5335648r) |
| TFM7_5335648-2 | TGGGCAACACATACTAAAGG |  |
| TFM8_5442376-1 | GACCGTTTAGGCAACAAGGA | Probe for Northern (5442335f, homolog of ncl0160) |
| TFM8_5442376-2 | AACAAGAAGCACGCGATATG |  |
| TFM9_6178702-1 | TTCCAAGTTATAGGATTACTGC | Probe for Northern (6178702f) |
| TFM9_6178702-2 | GGTTGCTGTTGCTGTGGTG |  |
| Nc6-2f | TCTGGATCATACTCATAGCCG | Probe for Northern (NsiR3) |
| Nc6-3r | TCAAACGAAATGGCGAGATG |  |
| 2050703-2 | CATCTGCCTCTGCCTCTTCTG | Probe for Northern (2050703f) |
| 2050703-3 | CCTTCCTTGTAGGCAGTCGAG |  |
| NC-T1-2 | TTTGGTGTTCCCTAAAACTGG | Probe for Northern (268249f) |
| NC-T1-3 | GCAAATGGCGGTTTGGTAAAT |  |
| NC-T3-2 | CCAAACTTAATGGGTAATGACTCG | Probe for Northern (2462444f) |
| NC-T3-3 | CGAATGTGGGCTTGTCTGTA |  |
| Nc7-TESTf | AATTGTGAGGATAGAAATGATTAAC | Probe for Northern (4547556r, NsiR8) |
| Nc7-TESTr | GTTTTTTCTAGATTAAGGCAATTTAATCCG |  |
| NC-7-6 | TTGGCTCACCACCAAACCTCTCTATAAATCC | Primer extension and Northern (4547237r) |
| NC-T10-3 EcoRI | GAATTCGGCAATTGTGAATTGCG | Probe for Northern (3797603f) |
| petE/T10 | TAAAATGAAATAACACAGCCGACACCTTCAC |  |
