# Supplementary material for: Identification of Conserved and Potentially Regulatory Small RNAs in Heterocystous Cyanobacteria
Source: Front Microbiol. 2016 Feb 1;7:48. doi: 10.3389/fmicb.2016.00048 (PMC4734099; doi:10.3389/fmicb.2016.00048)
Supplement: Supplementary file 2 [file Table2.DOCX]

**Table S2.** Genomes analyzed and the identifiers used throughout this work^a^.

|  | **Organism** | **ID** |  |  | **Organism** | **ID** |
| --- | --- | --- | --- | --- | --- | --- |
|  | *Trichodesmium erythraeum* IMS101 | Tric_IMS101 |  |  | *Cyanothece* sp. ATCC 51472 | Cyano_51472 |
|  | *Arthrospira platensis* str. Paraca | Arth_Paraca |  |  | *Cyanothece* sp. ATCC 51142 | Cyano_51142 |
|  | *Arthrospira platensis* NIES-39 | Arth_NIES39 |  |  | *Crocosphaera watsonii* WH0003 | Croc_WH0003 |
|  | *Arthrospira* sp. PCC 8005 | Arthro_8005 |  |  | *Crocosphaera watsonii* WH8501 | Croc_WH8501 |
|  | *Arthrospira maxima* CS-328 | Arth_CS-328 |  |  | *Cyanothece* sp. PCC 8802 | Cyanot_8802 |
|  | *Oscillatoria nigro-viridis* PCC 7112 | Oscill_7112 |  |  | *Cyanothece* sp. PCC 8801 | Cyanot_8801 |
|  | *Microcoleus vaginatus* FGP-2 | Micro_FGP-2 |  |  | *Synechocystis* sp. PCC 6803 | Synech_6803 |
|  | *Oscillatoria* sp. PCC 6506 | Oscill_6506 |  |  | *Pleurocapsa* sp. PCC 7327 | Pleuro_7327 |
|  | *Oscillatoria formosa* PCC 6407 | Oscill_6407 |  |  | *Microcystis aeruginosa* NIES-843 | Mic_NIES843 |
|  | *Oscillatoria* sp. PCC 10802 | Oscil_10802 |  |  | *Cyanothece* sp. PCC 7822 | Cyanot_7822 |
|  | *Oscillatoria acuminata* PCC 6304 | Oscill_6304 |  |  | *Cyanothece* sp. PCC 7424 | Cyanot_7424 |
|  | *Fischerella* sp. JSC-11 | Fisc_JSC-11 |  |  | *Gloeocapsa* sp. PCC 73106 | Gloeo_73106 |
|  | *Fischerella thermalis* PCC 7521 | Fische_7521 |  |  | *Synechococcus* sp. PCC 7002 | Synech_7002 |
|  | *Fischerella muscicola* PCC 7414 | Fische_7414 |  |  | *Leptolyngbya* sp. PCC 7376 | Leptol_7376 |
|  | *Fischerella* sp. PCC 9431 | Fische_9431 |  |  | *Cyanobacterium stanieri* PCC 7202 | Cyanob_7202 |
|  | *Fischerella muscicola* SAG1427-1 | F_SAG1427-1 |  |  | *Geminocystis herdmanii* PCC 6308 | Gemino_6308 |
|  | *Fischerella* sp. PCC 9339 | Fische_9339 |  |  | *Cyanobacterium aponinum* PCC 10605 | Cyano_10605 |
|  | *Fischerella* sp. PCC 9605 | Fische_9605 |  |  | *Stanieria cyanosphaera* PCC 7437 | Stanie_7437 |
|  | *Cyanobacterium* PCC 7702 | Cyanob_7702 |  |  | *Pleurocapsa* sp. PCC 7319 | Pleuro_7319 |
|  | *Chlorogloeopsis fritschii* PCC 6912 | Chloro_6912 |  |  | *Xenococcus* sp. PCC 7305 | Xenoco_7305 |
|  | *Chlorogloeopsis* sp. PCC 9212 | Chloro_9212 |  |  | *Microcoleus* sp. PCC 7113 | Microc_7113 |
|  | *Mastigocladopsis repens* PCC 10914 | Masti_10914 |  |  | *Crinalium epipsammum* PCC 9333 | Crinal_9333 |
|  | *Calothrix* sp. PCC 7103 | Caloth_7103 |  |  | *Chamaesiphon minutus* PCC 6605 | Chamae_6605 |
|  | *Calothrix* sp. PCC 6303 | Caloth_6303 |  |  | *Geitlerinema* sp. PCC 7105 | Geitle_7105 |
|  | *Nostoc* sp. PCC 7524 | Nostoc_7524 |  |  | *Synechococcus elongatus* PCC 7942 | Synech_7942 |
|  | *Nostoc* sp. PCC 7120 | Nostoc_7120 |  |  | *Synechococcus elongatus* PCC 6301 | Synech_6301 |
|  | *Anabaena variabilis* ATCC 29413 | Anaba_29413 |  |  | *Prochlorothrix hollandica* PCC 9006 | Prochl_9006 |
|  | *Nostoc* sp. PCC 7107 | Nostoc_7107 |  |  | *Leptolyngbya* sp. PCC 6406 | Leptol_6406 |
|  | *Cylindrospermum stagnale* PCC 7417 | Cylind_7417 |  |  | *Nodosilinea nodulosa* PCC 7104 | Nodosi_7104 |
|  | *Anabaena cylindrica* PCC 7122 | Anabae_7122 |  |  | *Leptolyngbya* sp. PCC 7375 | Leptol_7375 |
|  | *Anabaena* sp. PCC 7108 | Anabae_7108 |  |  | *Geitlerinema* sp. PCC 7407 | Geitle_7407 |
|  | *Nostoc azollae* 0708 | Nostoc_0708 |  |  | *Leptolyngbya boryana* PCC 6306 | Leptol_6306 |
|  | *Calothrix* sp. PCC 7507 | Caloth_7507 |  |  | *Thermosynechococcus elongatus* BP-1 | Thermo_BP-1 |
|  | *Microchaete* sp. PCC 7126 | Microc_7126 |  |  | *Synechococcus* sp. PCC 6312 | Synech_6312 |
|  | *Nodularia spumigena* CCY 9414 | Nodula_9414 |  |  | *Cyanothece* sp. PCC 7425 | Cyanot_7425 |
|  | *Nostoc punctiforme* PCC 73102 | Nosto_73102 |  |  | *Acaryochloris marina* MBIC11017 | A_MBIC11017 |
|  | *Scytonema hofmanni* UTEX 2349 | Scyton_2349 |  |  | *Acaryochloris* sp. CCMEE 5410 | A_CCMEE5410 |
|  | *Rivularia* sp. PCC 7116 | Rivula_7116 |  |  | *Synechococcus* sp. PCC 7502 | Synech_7502 |
|  | *Synechocystis* sp. PCC 7509 | Synech_7509 |  |  | *Pseudanabaena* sp. PCC 6802 | Pseuda_6802 |
|  | *Gloeocapsa* sp. PCC 7428 | Gloeoc_7428 |  |  | *Pseudanabaena biceps* PCC7 429 | Pseuda_7429 |
|  | *Chroococcidiopsis thermalis* PCC 7203 | Chrooc_7203 |  |  | *Pseudanabaena* sp. PCC 7367 | Pseuda_7367 |
|  | *Dactylococcopsis* salina PCC 8305 | Dactyl_8305 |  |  | *Synechococcus* sp. JA-3-3Ab | Syn_JA3-3Ab |
|  | *Halothece* sp. PCC 7418 | Haloth_7418 |  |  | *Synechococcus* sp. PCC 7336 | Synech_7336 |
|  | *Spirulina subsalsa* PCC 9445 | Spirul_9445 |  |  | *Gloeobacter violaceus* PCC 7421 | Gloeob_7421 |
|  | *Cyanothece* sp. CCY0110 | Cya_CCY0110 |  |  |  |  |

^a^Genomes are arranged and color-coded as in ([Shih et al., 2013](#_ENREF_33)) as follows: filamentous non-heterocystous in green, heterocystous ramified in blue, heterocystous non-ramified in pink, baeocystous in orange and unicellular in black. The reference organism is indicated in red.
