# Supplementary figures and images for: Identification of Conserved and Potentially Regulatory Small RNAs in Heterocystous Cyanobacteria
Source: Front Microbiol. 2016 Feb 1;7:48. doi: 10.3389/fmicb.2016.00048 (PMC4734099; doi:10.3389/fmicb.2016.00048)

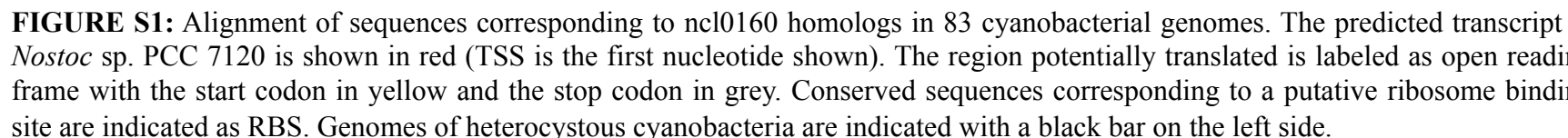

Supplement: Supplementary file 4 [file Image1.PDF]
