# Supplementary material for: Identification of Conserved and Potentially Regulatory Small RNAs in Heterocystous Cyanobacteria
Source: Front Microbiol. 2016 Feb 1;7:48. doi: 10.3389/fmicb.2016.00048 (PMC4734099; doi:10.3389/fmicb.2016.00048)
Supplement: Supplementary file 5 [file Image2.PDF]

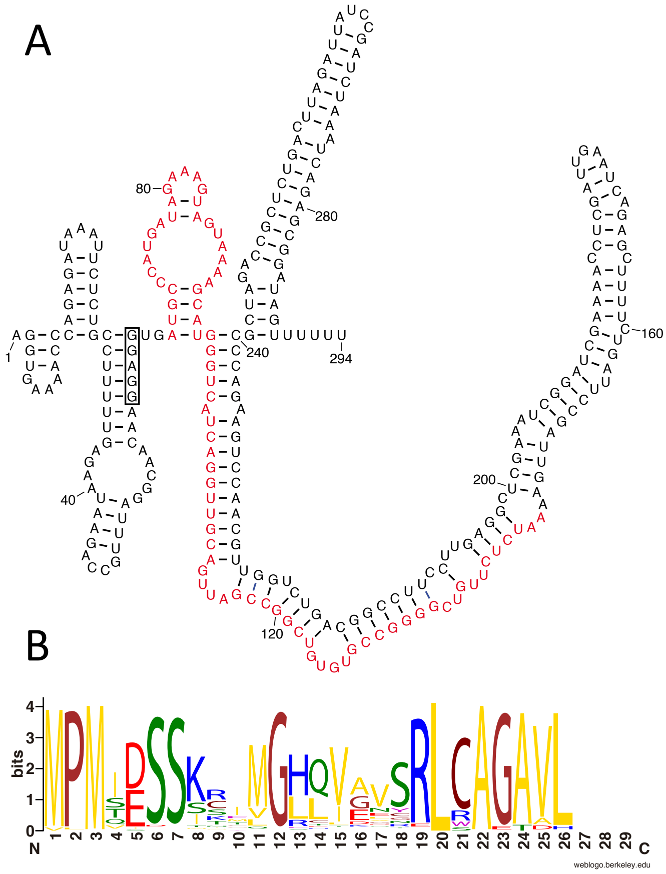

Figure S2. Structure of the ncl0160 homolog from *Nostoc* sp PCC 7120 and consensus sequence of the predicted encoded peptide. (A) Secondary structure of the ncl0160 homolog in *Nostoc* sp. PCC 7120 as predicted by mLocARNA (Will et al., 2012) from a set of 27 heterocyst-forming cyanobacteria included in this study, with modifications. A putative Shine-Dalgarno sequence is boxed. The potentially translated region is indicated in red. (B) Web logo (Crooks et al., 2004) of the peptide that could be translated from the ncl0160 homologs in 83 cyanobacterial genomes. The color code for amino acids is as follows; gold (hydrophobic), red (positively charged), blue (negatively charged), green (polar, no charge), maroon (proline and glycine), orange (cysteine) and purple (aromatic).
