# Supplementary material for: Identification of Conserved and Potentially Regulatory Small RNAs in Heterocystous Cyanobacteria
Source: Front Microbiol. 2016 Feb 1;7:48. doi: 10.3389/fmicb.2016.00048 (PMC4734099; doi:10.3389/fmicb.2016.00048)
Supplement: Supplementary file 6 [file Image3.pdf]

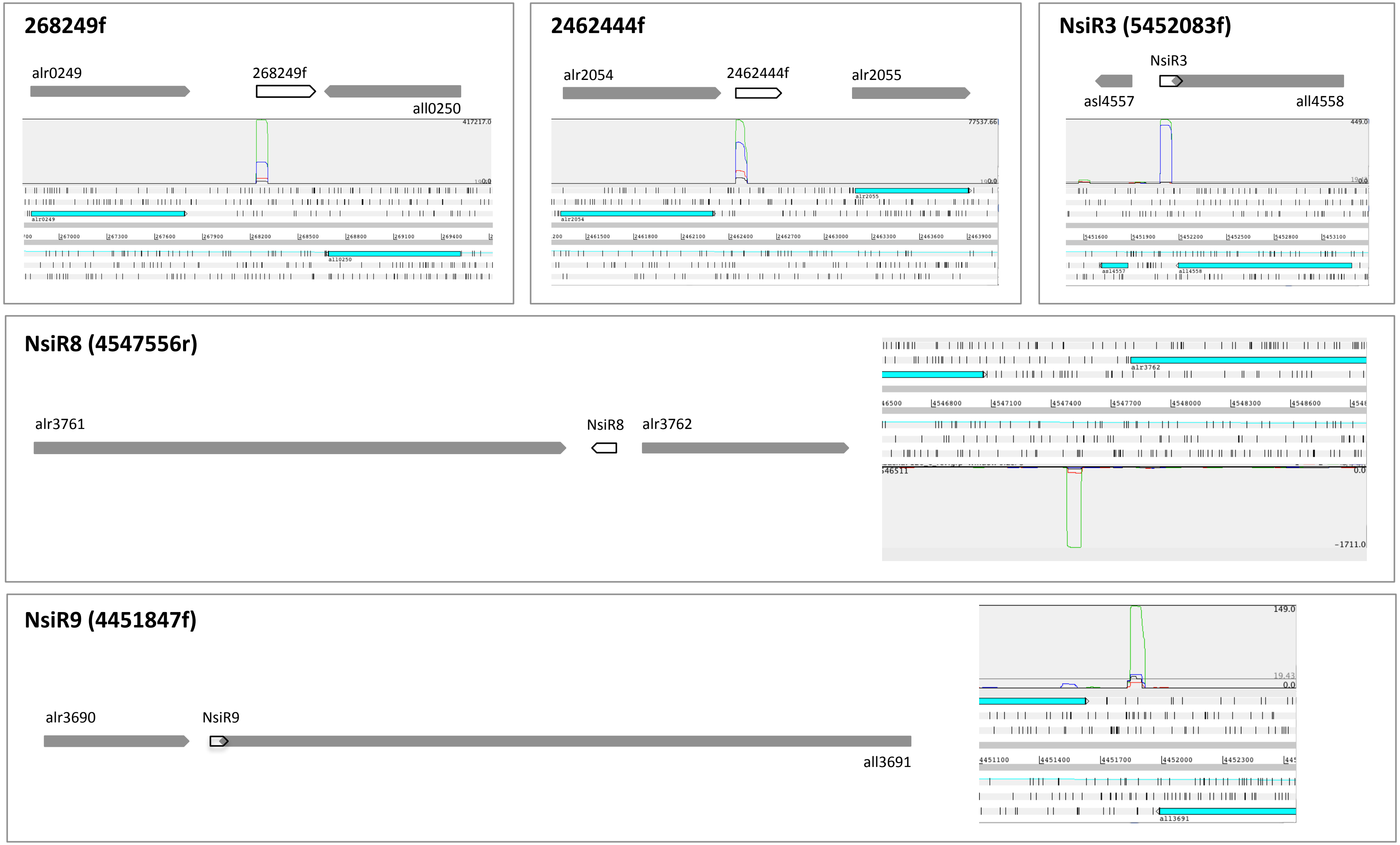


**FIGURE S3:** Genomic context and graphical presentation of the RNA-Seq reads associated to several sRNAs mentioned in the text. In the schematic representation of the contexts, the sRNAs are shown in white and the open reading frames are shown in grey. The RNA-Seq reads (Mitschke et al., 2011b) are shown in combination with a scheme of the genome generated with Artemis (Rutherford et al., 2000) that includes the open reading frames in turquoise. Reads are color-coded as follows: PCC7120 wild type strain in red ($NH_4^+$) and green (8 h $N_2$), *hetR* mutant in black ($NH_4^+$) and blue (8 h $N_2$). Note that, because of the RNA-Seq protocol used, the reads are all ≤ 75 nucleotides long and correspond to the 5' ends of the transcripts only.
